# College and Hospital Notes

**Published:** 1899-02

**Authors:** 


### COLLEGE AND HOSPITAL NOTES.

THE Skin and Cancer Hospital, New York, recently held its annua meeting, at which the following officers were elected: President, J. Cleveland Cady; vice-president, William C. Witter; treasurer, Edward Winslow, No. 17 Nassau street, and secretary, Dr. L. Duncan Bulkley, No. 4 East Thirty-seventh street.

Reports were received from the committees and departments of the hospital, in relation to the work of the last year, which has been a notable one for the institution, on account of the completion of its new building, at Nineteenth street and Second avenue, built entirely from the proceeds of the real estate formerly held by the hospital.

The new hospital shows advancement in all those features that experts consider desirable in a hospital, combined with thorough economy at every point. The building itself is arranged with great ingenuity, so that with little walking one can readily see every part of it. Every room is well lighted; the ventilation is perfect, and its appointments are all so devised that they can easily be kept clean.

The hospital has about sixty beds. A house in Nineteenth street, connected with the hospital furnishes accommodations for the employees. There is a dispensary on the first floor and a complete system of baths of various kinds in the basement. The building is fire-proof throughout and substantially built.

The work of this hospital is to a large extent for the relief of those who are completely disabled and thrown out of the condition of self-support by maladies which have not impaired their strength or general health.

The hospital has had great success in treating cases of cancer. This disease has many distressing forms that are amenable to expert surgical treatment, as the records of this hospital have shown.

The hospital is entirely dependent upon the public for its support, its property being wholly comprised in the new building and its plant. It is run with great economy and care, so that there are few objects for which a given amount of money small, or large, can be applied so directly to the result desired, or that will render so many helpless people helpful and independent for the money expended. Its work has fully justified its motto of "helping people to help themselves."

---

ANOTHER generous gift has been made to a medical college in this city. The sum of $50,000 has been given to Columbia University for the purpose of establishing free beds for children in the Roosevelt Hospital. The beds are to be dedicated to the name of Professor Abraham Jacobi. This is another gratifying evidence of interest in the endowment of medical schools. No one deserves the recognition of a long service as a distinguished teacher more than Dr. Jacobi. —*Post Graduate*, New York.

---

CORNELL UNIVERSITY, the first college to admit women in co-education with men, has also been obliged on adding a medical department to allow the sexes to study together. The professors it is stated were at first opposed to receiving the young women in the same medical classes with the young men, but the charter of the institution having been granted for co-education, it was not possible to close any department to either sex. The example of the Medical Department of the University of Buffalo, which admitted women in 1875 on the same basis as men, is thus followed. Nineteen young women are enrolled among the medical students of Cornell.

---

THE number of matriculates attending the New York Post-Graduate Medical School and Hospital on November 22, 1898, was 117, one more than on the same date in 1897. The number of patients in the hospital was 143, 105 of whom were free. During the past year gifts for the maintenance of beds in perpetuity have been made to the Post-Graduate Hospital by Dr. George N. Miller, The Orford Copper Company, Mr. and Mrs. Leonard Lewisohn, "In memory of Samuel Lewisohn;" Mr. and Mrs. Jefferson Hogan, "In memory of Jefferson Hogan, Jr." The perpetual support of a bed in the orthopedic ward has also been commenced by Mrs. Robert M. Thompson. We glean these interesting data from a recent number of the *Post-Graduate.*

---

On the recommendation of the Medical faculty and senatus of the University of Edinburgh, and with the permission of the University Court, Dr. J. W. Ballantyne, lecturer on midwifery and gynecology in the Medical College for Women, Minto House, will deliver a series of six lectures on teratology and antenatal pathology within the University (New Medical Buildings). The lectures, which are free to the members of the medical profession, will be given in the medical jurisprudence class-room, at 5 o'clock p. m., on consecutive Fridays in February and March, 1899.

